# Changes in Thyroid and Glycemic Status and Food Intake in Children with Excess Weight Who Were Submitted for a Multi-Component School Intervention for 16 Months

**DOI:** 10.3390/ijerph17113825

**Published:** 2020-05-28

**Authors:** Vinicius J. B. Martins, Andrea R. Filgueiras, Viviane B. P. Almeida, Rúbia C. S. de Moraes, Ana L. Sawaya

**Affiliations:** 1 Department of Physiology and Pathology, Federal University of Paraíba, Health Sciences Center, Campus I, Cidade Universitária, João Pessoa 58051-900, Brazil; 2 Department of Physiology, Federal University of São Paulo, Rua Botucatu, 862, Edifício de Ciências Biomédicas, 2 andar, São Paulo 04023-060, Brazil; andrea.filgueiras@gmail.com (A.R.F.); vivisbell@hotmail.com (V.B.P.A.); al.sawaya@yahoo.com.br (A.L.S.); 3 Department of Nutrition, Federal University of Paraíba, Health Sciences Center, Campus I, Cidade Universitária, João Pessoa 58051-900, Brazil; rubiacartaxo@gmail.com

**Keywords:** thyroid status, weight excess, insulin, food consumption, weight excess treatment

## Abstract

We investigated if children with excess weight who submitted to two types of intervention at school for 16 months showed improvements in thyroid and glycemic function and food intake. Children (8–11 years) with a body mass index-for-age (BMI/A) of ≥1 Z score were divided into two groups: Treatment 1 (*n* = 73) involved motivation to adopt healthier lifestyle; Treatment 2 (*n* = 103) involved performing weekly nutritional education, motivational, and physical activities at school. A semi-quantitative food frequency questionnaire was used. The delta BMI/A were similar after 16 months; Treatment 1 showed higher decrease in thyroid-stimulating hormone (TSH; median (range)): −0.45 (−3.19 to 2.17) and 0.06 (−4.57 to 1.63) mIU/L, *p* = 0.001), FreeT3 (−0.46 (−2.92 to 1.54) and −0.15 (−2.46 to 1.38) pmol/L, *p* = 0.038), and FreeT4 −1.41 (−6.18 to 3.47) and −0.90 (−4.89 to 2.96) pmol/L, *p* = 0.018), followed by decrease in energy intake (7304 (6806 to 7840) and 8267 (7739 to 8832) kJ, *P*treatment = 0.439, *P*time <0.001, interaction group–time *p* < 0.001), macronutrients and sugar. A positive correlation between FreeT3 and BMI/A, and a negative correlation with FreeT4 and insulin were found at baseline (r 0.212, *p* < 0.01; r −0.155, *p* < 0.01, respectively) and follow-up (r 0.222, *p* < 0.01; r −0.221, *p* < 0.01). The decrease in overall diet and particularly sugar intake was accompanied by a greater reduction in TSH and FreeT3 in Treatment 1, demonstrating the impact of dietary intake on thyroid function.

## 1. Introduction

Changes in thyroid function have been described in children [1,2] and adults with obesity [3]. These studies usually report an increase in the thyroid-stimulating hormone (TSH) [4], free T3 (FT3), and/or free T4 (FT4) concentrations [5,6]. It remains unclear whether these changes could represent a beneficial adaptive process to increase total energy expenditure in an attempt to reduce body weight [5], similar to the physiological adaptations of thyroid hormones in response to undernutrition [7]. On the contrary, an increase in TSH concentration has also been found without alteration in the FT4 and FT3 concentration [3,8], which characterizes subclinical hypothyroidism [9]. This condition is highly prevalent among obese patients than among controls. It is associated with increased low-density lipoproteins (LDL) cholesterol and fatal strokes [9] and is less adaptive in relation to increased thyroid hormones.

Another consequence of obesity in children and adolescents is insulin resistance, which is associated with the development of metabolic and cardiovascular diseases in early life [10,11]. Insulin resistance can reduce the activity of deiodinase 2 in the thyrotrophic cells, an enzyme that converts T4 into T3, resulting in an increase in the TSH concentration in obese individuals [12]. Brufani et al. [13] evaluated obese children and found that TSH and FT4 concentrations were associated with reduced insulin sensitivity. In this line, the hypothalamus–pituitary–thyroid and insulin axes are interrelated and show impaired function in obesity.

Weight excess affects 340 million children and adolescents worldwide [14]. In addition to the deleterious effects on health [15], weight excess is associated with a high financial impact on the health system [16]. Obesity is difficult to reverse [17], and multi-professional strategies are recommended, including dietary and lifestyle changes with frequent physical exercise [18]. It has been described that changes in thyroid function and structure are reversed after clinical treatment provided to obese children and adolescents [19]. Based on these findings, we hypothesize that treatment at school, including all students of the same classroom, could promote improvements in thyroid hormones, food intake, and body mass index for age (BMI/A) in children with excess weight. The treatment performed directly at school can guarantee better attendance and surveillance compared with the standard clinical treatment. In addition, treating all children (with or without weight excess) could contribute to the overall dietary and lifestyle changes and avoid stigmatization of the weight excess children.

The present study aims to investigate whether weight excess children who have been submitted to two multicomponent interventions in the school environment for 16 months would show improvements in thyroid and glycemic functions, food intake, and BMI/A. The study also aims to evaluate whether a higher FT3 concentration is associated with higher weight loss after 16 months of intervention and whether thyroid hormones correlate with anthropometric and metabolic variables in the baseline and follow-up.

## 2. Materials and Methods

### 2.1. Population and Study Design

**Figure 1:**
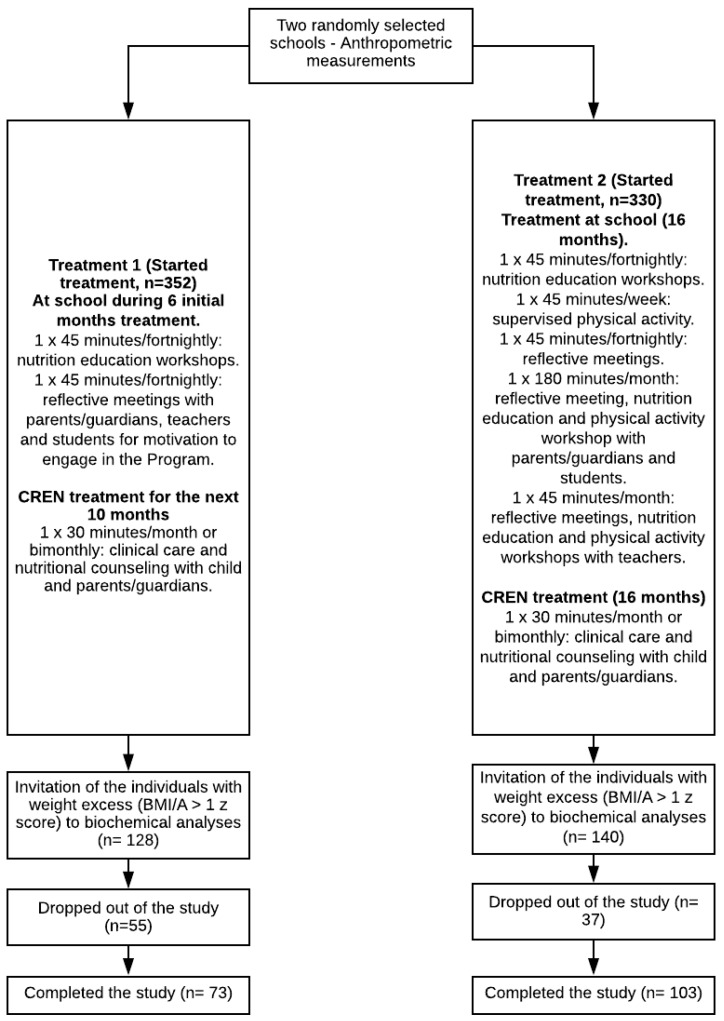
Flowchart of the study design. CREN, Centre for Nutritional Recovery and Education.

This study was conducted in two public schools in the city of São Paulo, Brazil. Three schools were eligible, and two of them were randomly selected to receive Treatment 1 or 2 (Figure 1). First, the weight and height of male and female children aged 8–11 years were measured to identify which children were overweight or obese (BMI/A ≥ 1 Z score and normal height for age (HAZ > −2)) in the two schools. Results of anthropometric assessment at School 1 (Treatment 1) showed that 36% of children had weight excess, of whom 20% were overweight and 16% were obese. In School 2 (Treatment 2), 42% of children had weight excess, of whom 25% were overweight and 17% were obese. At this time, no biochemical examination was performed, and the treatment was applied in all children who participated in the classroom activities. All overweight/obese children who were eligible for the study underwent blood collection and biochemical analyses in their respective schools (Figure 1).

Treatment 1 consisted of weekly meetings held in the classroom during the first six months to motivate the adoption of a healthy lifestyle. These meetings were conducted by nutritionists and psychologists. Three meetings were held with parents and teachers to integrate and motivate parents/guardians to change their eating behavior and promote a healthy lifestyle. After this period, all students with excess weight were referred for outpatient treatment at the Centre for Nutritional Recovery and Education (CREN) and followed for 10 months. This treatment was considered to be the control treatment. After six months of motivational workshops at school, weight excess children were invited for outpatient follow-up. Treatment 2 involved a fortnightly theoretical/practical group receiving nutritional education and performing reflective activity as well as weekly physical activity. The activities took place at school and were carried out in the classroom with the whole class for 16 months with the supervision of a psychologist, a nutritionist, and a physical educator. The teachers participated in the entire program as collaborators, favoring the participation of children in the proposed activities. Specific monthly meetings were also held for teachers and parents/guardians. Weight excess children received outpatient care at CREN. Previous studies indicated that longer than one year and high-intensity treatments for weight excess were more effective than short-term interventions [20]. The study lasted 16 months following the school schedule. This study was registered in the Brazilian Registry of Clinical Trials (ReBEC Primary ID number: RBR-9t2jr8).

Individuals with neurological, cardiovascular, respiratory, or metabolic disorders, those who reported previous use of anabolic steroids or psychotropic drugs, twins, and pregnant women were excluded from the study.

The study was approved by the Ethics Committee of the Federal University of São Paulo (CAAE: 34.304.714.40000.5505), in accordance with the national laws, and all procedures were conducted in accordance with the principles of the Helsinki Declaration.

### 2.2. Anthropometry

The children were weighed on a portable digital scale, with a precision of 100 g and a capacity of 150 kg (Plenna^®^ MEA 07400, São Paulo, Brazil). Height was measured with a portable stadiometer with an accuracy of 1 mm (Alturaexata^®^, Belo Horizonte, Brazil). HAZ and BMI/A were determined using the World Health Organization (WHO) curves as standards. The WHO AnthroPlus program (v. 1.0.4, Geneva, Switzerland) was employed to assess nutritional status.

A measuring tape with 1 mm of precision was used to measure the waist and hip circumference. The measurements were obtained with the children in standing position, keeping the abdomen and the arms relaxed beside the body. For the waist circumference, the measuring tape was placed at the midpoint between the last rib and the iliac crest, and the hip was measured over the greater trochanter. The waist-to-hip ratio (WHR) was calculated as the ratio of these circumferences, and the same was performed to measure the waist-to-height ratio (WHtR).

### 2.3. Biochemistry

Blood samples were obtained after a 10-h night fast to measure the glucose, insulin, TSH, FT4, and FT3 levels. These assays were performed by chemiluminescence in the Research Incentive Fund Association (AFIP, in Portuguese). The normal values were 0.34−6.0 mIU/L for TSH, 6.95−20.59 pmol/L for FT4, and 3.84−5.97 pmol/L for FT3. The homeostasis model assessment (HOMA-IR) was performed to measure insulin resistance using the following formula [21]: HOMA-IR = fasting insulin (mU/L) × fasting glucose (mmol/L)/22.5. Blood samples were collected at the beginning of the study and after 16 months.

### 2.4. Dietary Evaluation

The dietary evaluation was performed using the 88-item semi-quantitative food frequency questionnaire [22]. To assist in the application of the questionnaire and determine the size of the portion, a photographic manual was used with images representing the portion sizes of each item [23]. The complete description of the application of this questionnaire is described by Filgueiras et al. [24].

### 2.5. Statistical Analysis

The variables were analyzed using the Shapiro–Wilk normality test. Anthropometric variables were analyzed using Student’s *t*-test or the Mann–Whitney U test, whichever is appropriate. Biochemical variables (except glucose) were log-transformed (Napierian) and analyzed using analysis of covariance (ANCOVA) adjusted for sex and age, and the geometric mean is presented. A mixed between–within-subject two-way adjusted for sex and age was applied to compare the pre- and post-interventions and to analyze the group factor. The correlations between anthropometric and biochemical variables were evaluated using Pearson or Spearman correlations. The hormones were categorized as below normal, normal, and above normal to analyze the distribution between groups using the chi-square test. For dietary intake analyses, comparisons between groups, times, and interactions were verified using the generalized estimating equations models. The analyses were performed using the SPSS for Windows (version 20.0; IBM Corporation, Armonk, NY, USA), with an alpha of 0.05.

## 3. Results

The anthropometric data obtained at the beginning of the study are summarized in Table 1. The age of children in Treatment 1 was significantly higher than that of children in Treatment 2. No differences were observed for HAZ, BMI/A, WHR, WHtR, and glucose. Children in Treatment 1 showed higher TSH, FT3, and FT4 and lower insulin concentration and HOMA-IR than those in Treatment 2. 

There were no changes in the delta of the anthropometric variables between the two types of interventions, but children in Treatment 1 showed a significant decrease in TSH, FT3, and FT4 compared with those in Treatment 2, while insulin, glucose, and HOMA-IR did not differ (Table 2).

At the beginning, no differences were observed in the prevalence of overweight and obesity between Treatment 1 (39 overweight children (21.8%) and 37 obese children (20.7%)) and Treatment 2 (59 overweight children (33%) and 44 obese children (24.6%); Pearson chi-square = 0.628, *p* = 0.428). After 16 months, 11 (6.6%) children in the Treatment 1 group showed improvements in BMI/A, 35 (21%) were overweight, and 25 (15%) were obese; in Treatment 2, 15 (9%) showed improvements in BMI/A, 43 (25.7%) were overweight, and 38 (22.8%) were obese (Pearson chi-square 0.385, *p* = 0.825).

A child in the Treatment 1 group showed TSH above normal at baseline and follow-up (baseline Fisher’s exact test (*p* = 0.415) and follow-up (*p* = 0.400)). All children showed normal FT4 at the beginning of the study and at follow-up.

**Figure 2:**
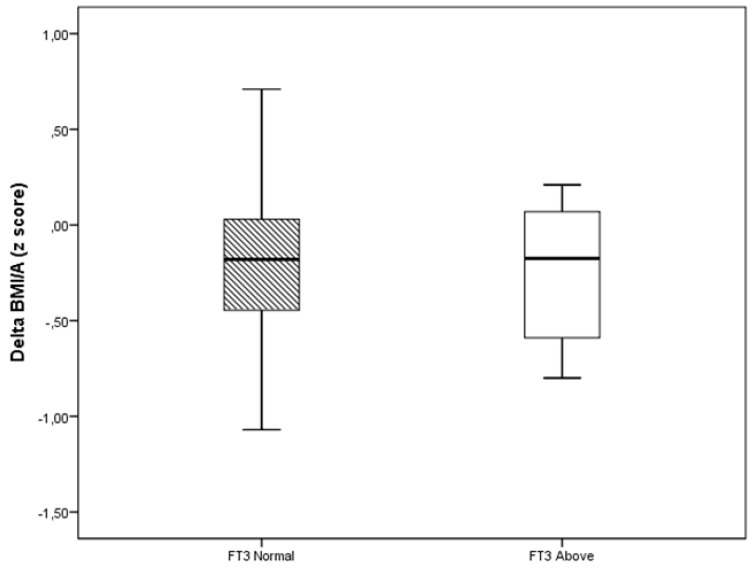
Delta of BMI/A (z score) based on the FT3 values at follow-up. The line represents the median (interquartile interval). Mann–Whitney test, *p* = 0.723.

The Treatment 1 group showed a higher proportion of children with FT3 above the reference values compared with the Treatment 2 group at baseline (Pearson chi-square = 20.24, *p* = < 0.001) and at follow-up (Fisher’s exact test, *p* = 0.014; Table 3). The delta of BMI/A, according to FT3 reference, regardless of the type of treatment, remained unchanged. Individuals with FT3 above normal value (n = 12) did not lose more BMI/A compared with those who had normal FT3 (n = 132) (Mann–Whitney test, *p* = 0.723; Figure 2).

**Figure 3:**
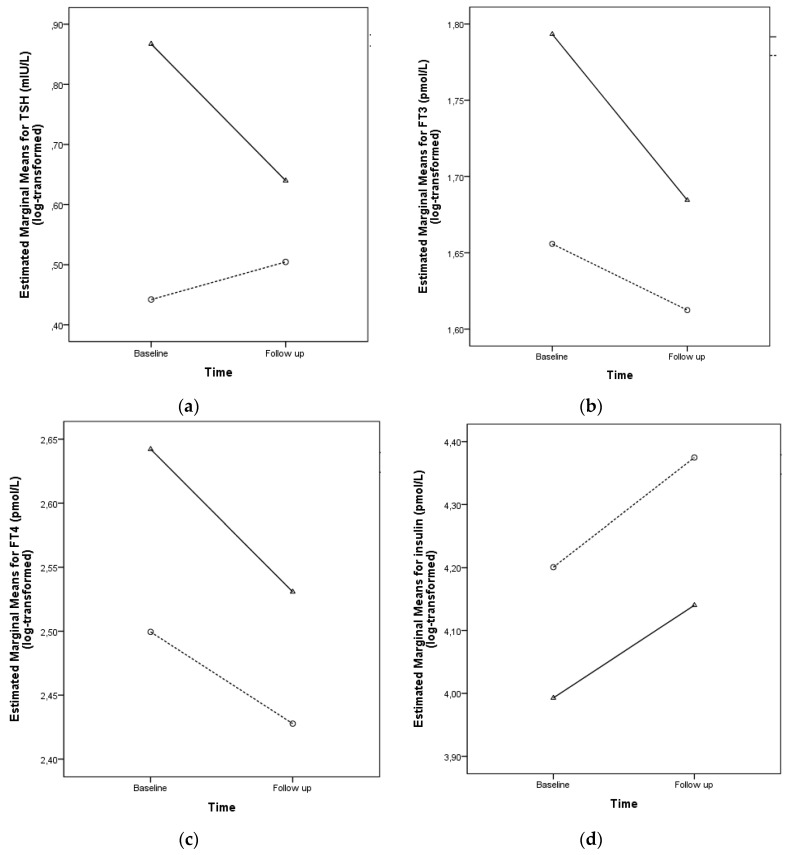
TSH, FT3, FT4, and insulin concentrations at baseline and follow-up after 16 months of treatment. Continuous line indicates Treatment 1, dashed line depicts Treatment 2. Mixed between–within-subject ANCOVA adjusted for age and sex. (**a**) TSH, main effect for time: Wilks’ λ = 0.986, F 1138 = 2.03, *p* = 0.156; Interaction between time and group: Wilks’ λ = 0.919, F 1138 = 12.18, *p* = 0.001; group factor: F = 13.62, *p* < 0.001. (**b**) FT3, main effect for time: Wilks’ λ = 1.000, F 1137 = 0.005, *p* = 0.943; interaction between time and group: Wilks’ λ = 0.963, F 1137 = 5.32, *p* = 0.023; group factor: F = 34.90, *p* < 0.001. (**c**) FT4, main effect for time: Wilks’ λ = 0.996, F 1137 = 0.504, *p* = 0.479; interaction between time and group: Wilks’ λ = 0.980, F 1137 = 2.73, *p* = 0.100; group factor: F = 39.68, *p* < 0.001. (**d**) Insulin, main effect for time: Wilks’ λ = 0.997, F 1139 = 0.435, *p* = 0.511; interaction between time and group: Wilks’ λ = 0.999, F 1139 = 0.09, *p* = 0.762; group factor: F = 9.57, *p* = 0.002.

TSH, FT3, FT4, and insulin concentrations were compared using mixed between–within-subject two-way ANCOVA (Figure 3). The Treatment 1 group showed higher concentrations of TSH compared with the Treatment 2 group (group factor *p* < 0.001). The Treatment 1 group showed lower TSH concentrations at follow-up, while the Treatment 2 group showed higher TSH concentrations (interaction factor *p* = 0.001; Figure 3A). The Treatment 1 group showed a stronger decrease in FT3 concentration, but higher values over time compared with the Treatment 2 group (Figure 3B). FT4 concentration did not decrease along with the treatment (*p* = 0.479), but the Treatment 1 group showed higher FT4 concentration over time (*p* < 0.001; Figure 3C). The Treatment 2 group showed higher insulin concentration compared with the Treatment 1 group (*p* = 0.002; Figure 3D).

Table 4 presents the data on energy, macronutrient, sodium, and sugar consumption in the study. Energy, protein, and sodium consumption decreased in the Treatment 1 group and increased in the Treatment 2 group. Fat consumption decreased over time in the Treatment 1 group but remained the same in the Treatment 2 group. Carbohydrate and sugar consumption decreased in both groups, with a greater decrease in the Treatment 1 group.

At the beginning of the study, a positive correlation was observed between TSH and sugar (Table 5). FT3 showed a positive correlation with glucose, BMI/A, and WHR. FT4 showed a negative correlation with insulin and a tendency with HOMA-IR. No correlation was found between insulin, TSH, and FT3. In the follow-up, no correlation was found between TSH and the other studied variables (Table 6). FT3 was positively correlated with BMI/A, WHR, and WHtR. Negative correlations were observed between FT4 with insulin, glycemia, and HOMA-IR.

## 4. Discussion

The high prevalence of weight excess among children is well recognized worldwide as well as the complexity of treatment and the need to maintain a healthy status. This condition includes changes in thyroid and insulin function and the development of comorbidities in early life, making obesity an important public health problem. The school has been considered as an important environment in the diagnosis and treatment of obesity [25]. In the present study, the treatment performed in school included meetings with parents and teachers, and their involvement was encouraged, as it is important for achieving an effective outcome [26]. 

The Treatment 1 group had a larger decrease in TSH, FT3, and FT4 concentrations the Treatment 2 group, following the greater reduction in the consumption of macronutrients, energy, sodium, and sugar, although the delta BMI/A after 16 months of treatment were similar between the two intervention groups. These findings showed that the differences in the diet, but not in the BMI/A delta, were associated with the higher decrease in TSH, FT4, and FT3 in the Treatment 1 group. Johannsen et al. [27] evaluated the metabolic adaptation in adults exposed to overfeeding with an additional 40% of the energy requirement (41% carbohydrate, 44% fat, and 15% protein) for eight weeks and found an increase in T3 concentration, but not TSH and T4, in the follow-up period. Studies conducted in rats fed with a high-fat diet for 18 or 24 weeks observed morphological changes and decreased in the concentration of total T4 [28,29].

The positive correlation between TSH and sugar at baseline may indicate that this hormone is particularly sensitive to sugar consumption. In addition, the decrease in sugar intake was higher in the Treatment 1 group than in the Treatment 2 group, along with a greater decrease in TSH, FT3, and FT4 concentrations. Strbak et al. [30] found that glucose intake promotes an additional increase in TSH concentration in volunteers aged 20 to 25 years who were exposed to a warm environment for 30 min, probably mediated by the dopaminergic system. On the contrary, the administration of glucose, sucrose, high corn syrup, or fructose in rats did not increase the thyrotropin-releasing hormone (TRH) expression in the ventromedial and lateral nucleus. However, compared with high amounts of corn syrup or fructose, the administration of sucrose increased the expression of this hormone in the paraventricular nucleus [31]. The mechanisms underlying these findings remain unclear. No correlations were found between any macronutrient and sodium with TSH, FT3, or FT4 concentrations.

Subclinical hypothyroidism can be defined as an increase in TSH concentration without alterations in FT4 [9]. Dahl et al. [8] found that weight excess boys, with BMI/A higher than 1.28 z score, had a higher prevalence of subclinical hypothyroidism than the control boys, and this difference was not observed in girls. In the present study, no differences were observed in the TSH concentrations between the two treatment groups, in both time points, and only one child presented with subclinical hypothyroidism.

The correlation of TSH and FT3 or FT4 with HOMA-IR has been described [32]. In the present study, a negative correlation was observed between T4 and HOMA-IR and insulin. Aeberli et al. [4] reported a decrease in TSH concentration in children who showed weight loss, and this was associated with improvements in insulin sensitivity. In Santos et al.’s study [33], children and adolescents with BMI/A of 3.0 z score were submitted for obesity treatment for a year to stimulate the adoption of a Mediterranean diet (carbohydrates (55%), proteins (15%), and lipids (30% total, with <10% saturated fat) and exercise. After treatment, no differences were found in thyroid function (TSH, FT4, and FT3), but HOMA-IR decreased among individuals who lost more than 0.5 BMI/A z score. A positive correlation was also observed between TSH, HOMA-IR, and BMI/A. In the present study, no correlations were observed between TSH and anthropometric indicators or HOMA-IR. 

The correlation between insulin and thyroid hormones can be explained, at least in part, by the effect of the thyroid hormones on the expression of GLUT4 in the muscle and adipose tissues. Compare with untreated rats, thyroidectomized rats showed a reduction in mRNA of GLUT4 in skeletal muscles, while treatment with FT3 increased the mRNA of GLUT4 [34]. One study found that women with severe hypothyroidism showed lower insulin sensitivity [35]. Thus, the higher concentration of thyroid hormones observed at the beginning of the study could be contributing to the lower HOMA-IR in the Treatment 1 group. The decrease in thyroid hormone concentration resulted in an increase in insulin concentration and HOMA-IR in the two treatment groups. One reason that can support this finding is the negative correlation between insulin and FT4 at the beginning of the study and at follow-up, although no correlation was found between TSH and FT3 with HOMA-IR in these two time points. These correlations may not have been detected because the TSH did not increase above the reference values. Lundbäck et al. [32] evaluated thyroid function, glucose, and insulin metabolism in obese children with an average age of 11 years. Children with high normal TSH had higher insulin resistance than those with low normal TSH. Moreover, FT3 was positively associated with insulin.

Increased thyroid function could indicate a metabolic adaptation to reduce body weight in excess weight individuals [36]. In this line, the concentrations of thyroid hormones usually decrease in undernourished individuals to prevent energy expenditure [37]. One study has suggested that the increase in thyroid hormone concentrations in weight excess individuals is more related to the deposit and maintenance of new tissues than to an increase in energy expenditure to promote weight loss [27]. This could explain, at least in part, the findings of the present study that, regardless of the group, higher concentrations of FT3 did not promote greater BMI/A loss.

A new finding of the present study is the increase in FT3 but normal TSH concentrations in comparison to the reference values. Studies have usually shown differences in mean values among obese children than in non-overweight control individuals, and these mean values were high normal, but within normal or slightly increased [5,38]. Leptin action in the hypothalamic nucleus increased the TRH and, consequently, TSH and T4/T3 concentrations [39]. However, leptin concentration was not evaluated in the present study. The increase in FT3 concentration may be related to the higher action of deiodinase [40] once the TSH and FT4 concentrations are within normal range.

Our study has some limitations. One of the limitations was the impossibility of following a typical randomization design, as only three schools were located close to the CREN to allow frequent outpatient attendance at the Centre. In addition, randomization was not performed because all children in the classrooms participated in the treatment activities, and all excess weight children were invited for biochemical analyses. Another limitation was the lack of control patients with weight excess, who can be used to compare the two treatment groups, given the impossibility of having controls without any intervention due to ethical issues. In order to work around this limitation, hormone concentrations were compared not only between the treated groups but also with reference values. Despite the statistical difference in age between the groups at baseline, the differences were physiologically small to promote substantial changes in the concentrations of TSH, FT3, FT4, and insulin. In addition, the analyses were adjusted for age and gender. However, the differences in the hormonal profile at baseline could be a confounding factor. To counteract these differences, we analyzed the delta obtained at follow-up and baseline, and used a mixed between–within-subject ANCOVA to compare the pre- and post-interventions.

## 5. Conclusions

The decrease in the delta BMI/A was similar in both treatment groups. The decrease in overall diet intake, in particular in sugar intake, was accompanied by a greater decrease in TSH and FT3 concentrations after Treatment 1, which demonstrates the impact of diet and, especially, sugar intake on thyroid function. The positive correlation between FT3 and BMI/A at the beginning and follow-up may indicate the process of adjusting the hormone concentration for the new body mass. Compared with the reference values, high FT3 concentrations did not promote additional BMI/A loss. In turn, the inverse relationship between FT4 and insulin, and the positive relationship between TSH and sugar may have a clinical impact and should be investigated in more detail.

## Figures and Tables

**Table 1:** Anthropometric and hormonal profile at baseline in both interventions ^a^.

Excess Weight (BMI ≥ 1 z Score)	Treatment 1 (n = 73; B 27, G46)	Treatment 2 (n = 103; B 51, G 52)	*p* ^b^
Age (years)	9.83 ± 0.85	9.40 ± 0.90	0.001
HAZ (z score)	0.60 ± 0.89	0.68 ± 0.93	0.557
BMI/A (z score)	2.04 ± 0.72	1.98 ± 0.70	0.588
Waist hip ratio	0.82 ± 0.04	0.82 ± 0.04	0.688
Waist-to-height ratio	0.49 ± 0.05	0.49 ± 0.04	0.949
TSH (mIU/L) ^†^	2.39 (2.12–2.69)	1.48 (1.34–1.62)	<0.001
FT3 (pmol/L) ^†^	5.93 (5.75–6.11)	5.26 (5.10–5.36)	<0.001
FT4 (pmol/L) ^†^	14.00 (13.59–14.29)	12.17 (11.93–12.42)	<0.001
Insulin (pmol/L) ^†^	54.03 (48.40–60.31)	65.99 (59.71–72.20)	0.008
Glucose (mmol/L)	5.14 ± 0.40	5.14 ± 0.33	0.903
HOMA-IR ^†^	2.05 (1.82–2.31)	2.50 (2.27–2.77)	0.014

^a^ Values are expressed as mean ± SD or mean (95% confidence interval). ^†^ Geometric means values; one-way analysis of covariance (ANCOVA) adjusted for sex and age. ^b^ *p* refers to *t*-test or ANCOVA. B, boys G, girls.

**Table 2:** Delta between baseline and follow-up of anthropometric and metabolic variables in both interventions ^a^.

Excess Weight (BMI ≥ 1 z Score)	Treatment 1 (n = 55, B18, G37)	Treatment 2 (n = 87, B43, G44)	*p* ^b^
HAZ (z score)	−0.02 (−0.80–1.00)	0.05 (−1.17–0.92)	0.156
BMI/A (z score)	−0.19 (−1.48–0.71)	−0.19 (−1.19–0.61)	0.717
Waist hip ratio	−0.01 (−0.07–0.06)	−0.01 (−0.09–0.09)	0.650
Waist-to-height ratio	−0.01 (−0.05–0.04)	−0.01 (−0.08–0.04)	0.081
TSH (mIU/L)	−0.45 (−3.19–2.17)	0.06 (−4.57–1.63)	0.001
FT3 (pmol/L)	−0.46 (−2.92–1.54)	−0.15 (−2.46–1.38)	0.038
FT4 (pmol/L)	−1.41 (−6.18–3.47)	−0.90 (−4.89–2.96)	0.018
Insulin (pmol/L)	8.13 (−91.62–80.82)	11.40 (−87.00–150.00)	0.688
Glucose (mmol/L)	−0.16 (−0.72–0.94)	−0.05 (−1.17–1.33)	0.227
HOMA-IR	0.20 (−3.79–3.10)	0.40 (−3.92–7.13)	0.473

^a^ Values are expressed as median (range). ^b^ Mann–Whitney test. B, boys G, girls.

**Table 3:** Distribution of individuals according to FT3 values ^a^.

**Type of Treatment**	**Baseline**			
	**FT3 Normal**	**FT3 Above**	**Total**	***p***
Treatment 1 n (%)	41 (23.3)	32 (18.1)	73 (41.4)	
Treatment 2 n (%)	89 (50.6)	14 (8.0)	103 (58.6)	0.001
Total	130 (73.9)	46 (26.1)	176 (100)	
**Type of treatment**	**Follow-up**			
	**FT3 Normal**	**FT3 Above**	**Total**	***p***
Treatment 1 n (%)	49 (34.0)	9 (6.2)	58 (40.3)	
Treatment 2 n (%)	83 (57.6)	3 (2.1)	86 (59.7)	0.014
Total	132 (91.7)	12 (8.3)	144 (100)	

^a^ n (% of total). Baseline: Pearson chi-square = 20.24; follow-up: Fisher’s exact test. Normal FT3 values: 3.84–5.99 pmol/L.

**Table 4:** Profile of total food intake before and after the intervention (n = 143).

Total	Baseline	16 Months	*p* Treatment	*p* Time	*p* Treatment × Time
**Energy (kJ)**					
Treatment 1	8575 (8116–9443)	7304 (6806–7840)	0.439	<0.001	<0.001
Treatment 2	8233 (7815–8677)	8267 (7739–8832)			
**Total Fat (g)**					
Treatment 1	77 (70–84)	62 (58–68)	0.163	0.002	<0.001
Treatment 2	74 (69–78)	74 (69–81)			
**Total carbohydrate (g)**					
Treatment 1	268 (251–287)	228 (211–246)	0.774	<0.001	0.004
Treatment 2	252 (239–265)	248 (233–264)			
**Total Protein (g)**					
Treatment 1	84 (77–91)	68 (64–73)	0.761	0.010	<0.001
Treatment 2	75 (71–79)	78 (73–84)			
**Sodium (mg)**					
Treatment 1	3315 (3056–3595)	2746 (2590–2911)	0.176	0.004	<0.001
Treatment 2	3150 (2998–3310)	3217 (2972–3482)			
**Sugar (g)**					
Treatment 1	107 (98–117)	84 (75–95)	0.078	<0.001	0.007
Treatment 2	89 (82–96)	83 (77–90)			

General estimated equations.

**Table 5:** Correlations between hormones and anthropometric data at baseline ^a^.

Variables	Age (Years)	TSH (mIU/L)	FT3 (pmol/L)	FT4 (pmol/L)	Insulin (pmol/L)	Glucose (mmol/L)	HOMA-IR	HAZ (z Score)	BMI/A (z Score)	WHR	Waist to Height Ratio
TSH (mIU/L) ^†^	−0.027										
FT3 (pmol/L)^†^	−0.152 *	0.279 **									
FT4 (pmol/L) ^†^	−0.020	0.333 *	0.364 **								
Insulin (pmol/L) ^†^	0.053	0.075	0.114	−0.155 *							
Glucose (mmol/L)	0.082	0.108	0.191 *	0.078	0.307 **						
HOMA-IR ^†^	0.061	0.085	0.133	−0.138	0.992 **	0.424 **					
HAZ (z score)	−0.088	−0.110	0.066	−0.128	0.273**	0.004	0.264 **				
BMI/A (z score)	−0.051	0.087	0.212 **	0.047	0.463 **	0.116	0.460 **	0.311 **			
WHR	−0.236 **	0.103	0.104	0.041	0.201 **	0.031	0.195 *	−0.037	0.431 **		
Waist to height ratio	−0.077	0.139	0.230 **	0.071	0.451 **	0.083	0.443 **	0.037	0.844 **	0.700 **	
Sugar (mg)	−0.039	0.183 *	0.124	0.034	−0.087	−0.083	−0.101	0.044	0.066	0.057	0.011

TSH, thyroid-stimulating hormone; FT3, free triiodothyronine; FT4, free thyroxine; HOMA-IR, homeostasis model assessment of insulin resistance; HAZ, height for age; BMI/A, BMI for age; WHR, waist:hip ratio. ^a^ Pearson correlation values; Spearman for sugar. ^†^ Log-transformed values of TSH, T3, T4, insulin, and HOMA-IR. * *p* < 0.05, ** *p* < 0.01.

**Table 6:** Correlations between hormones and anthropometric data at follow-up ^a^.

Variables	Age (Years)	TSH (mIU/L)	FT3 (pmol/L)	FT4 (pmol/L)	Insulin (pmol/L)	Glucose (mmol/L)	HOMA-IR	HAZ (z Score)	BMI/A (z Score)	WHR	Waist to Height Ratio
TSH (mIU/L) ^†^	0.085										
FT3 (pmol/L) ^†^	−0.227 **	0.118									
FT4 (pmol/L) ^†^	−0.030	0.119	0.270 **								
Insulin (pmol/L) ^†^	0.119	0.079	0.041	−0.221 **							
Glucose (mmol/L)	−0.015	0.137	−0.053	−0.209 *	0.298 **						
HOMA-IR ^†^	0.067	0.062	0.006	−0.260 **	0.922 **	0.373 **					
HAZ (z score)	−0.126	−0.104	0.092	−0.140	0.318 **	0.149	0.274 **				
BMI/A (z score)	−0.012	0.042	0.222 **	0.085	0.429 **	−0.028	0.364 **	0.235 **			
WHR	−0.251 **	0.068	0.182 *	0.065	0.190 *	−0.023	0.164 *	−0.069	0.420 **		
Waist to height ratio	0.011	0.123	0.250 **	0.151	0.407 **	−0.055	0.345 **	−0.043	0.887 **	0.677 **	
Sugar (mg)	0.001	0.058	−0.008	0.044	−0.157	−0.134	−0.187	0.002	−0.075	−0.001	−0.095

TSH, thyroid-stimulating hormone; FT3, free triiodothyronine; FT4, free thyroxine; HOMA-IR, homeostasis model assessment of insulin resistance; HAZ, height for age; BMI/A, BMI for age; WHR, waist:hip ratio. ^a^ Pearson correlation values; Spearman for sugar. ^†^ Log-transformed values of TSH, T3, T4, insulin, and HOMA-IR. * *p* < 0.05, ** *p* < 0.01.
